# Plasmolyzed yeast cell system for the encapsulation of *Pulicaria odora* polyphenols: RSM-based process optimization and functional characterization

**DOI:** 10.1007/s10068-026-02128-6

**Published:** 2026-03-25

**Authors:** Djamel Eddine Laib, Imen Laib, Hamdi Bendif, Sulaiman A. Alsalamah, Tarek H. Taha, Fehmi Boufahja, Walid Elfalleh, Stefania Garzoli

**Affiliations:** 1 Department of Agronomic Sciences, Faculty of Sciences, August 20, 1955 University, Skikda, Algeria; 2 Laboratory of the Optimization of Agricultural Production in Subhumid Areas, Faculty of Sciences, University 20 August 1955, 21000 Skikda, Algeria; 3 Department of Biology, College of Science, Imam Mohammad Ibn Saud Islamic University (IMSIU), 11623 Riyadh, Saudi Arabia; 4 Department of Chemistry and Technologies of Drug, Sapienza University, P. Le Aldo Moro, 5, 00185 Rome, Italy

**Keywords:** Polyphenols, Yeast encapsulation, Response surface methodology

## Abstract

This study focused on optimizing the encapsulation of polyphenols extracted from *Pulicaria odora* into plasmolyzed *Saccharomyces cerevisiae* yeast cells using RSM. The polyphenols were obtained via ethanolic maceration, and encapsulation performance was evaluated by varying two key factors: the yeast-to-extract ratio and incubation time. A CCD was employed to model the process and determine optimal conditions. The model predicted maximum encapsulation efficiencies of 95.83% for total polyphenols and 96.83% for total flavonoids. The microcapsules obtained under these optimized conditions were further characterized to evaluate their structural, chemical, and functional properties. SEM confirmed the incorporation of bioactives into the yeast matrix, while FTIR indicated molecular interactions between polyphenols and yeast cell wall components. DSC revealed enhanced thermal stability of the encapsulated forms. Antioxidant activity was significantly improved after encapsulation. The findings highlight the potential of plasmolyzed yeast cells as natural, biocompatible carriers for stabilizing and delivering plant-derived bioactives for several applications.

**Graphical abstract:**

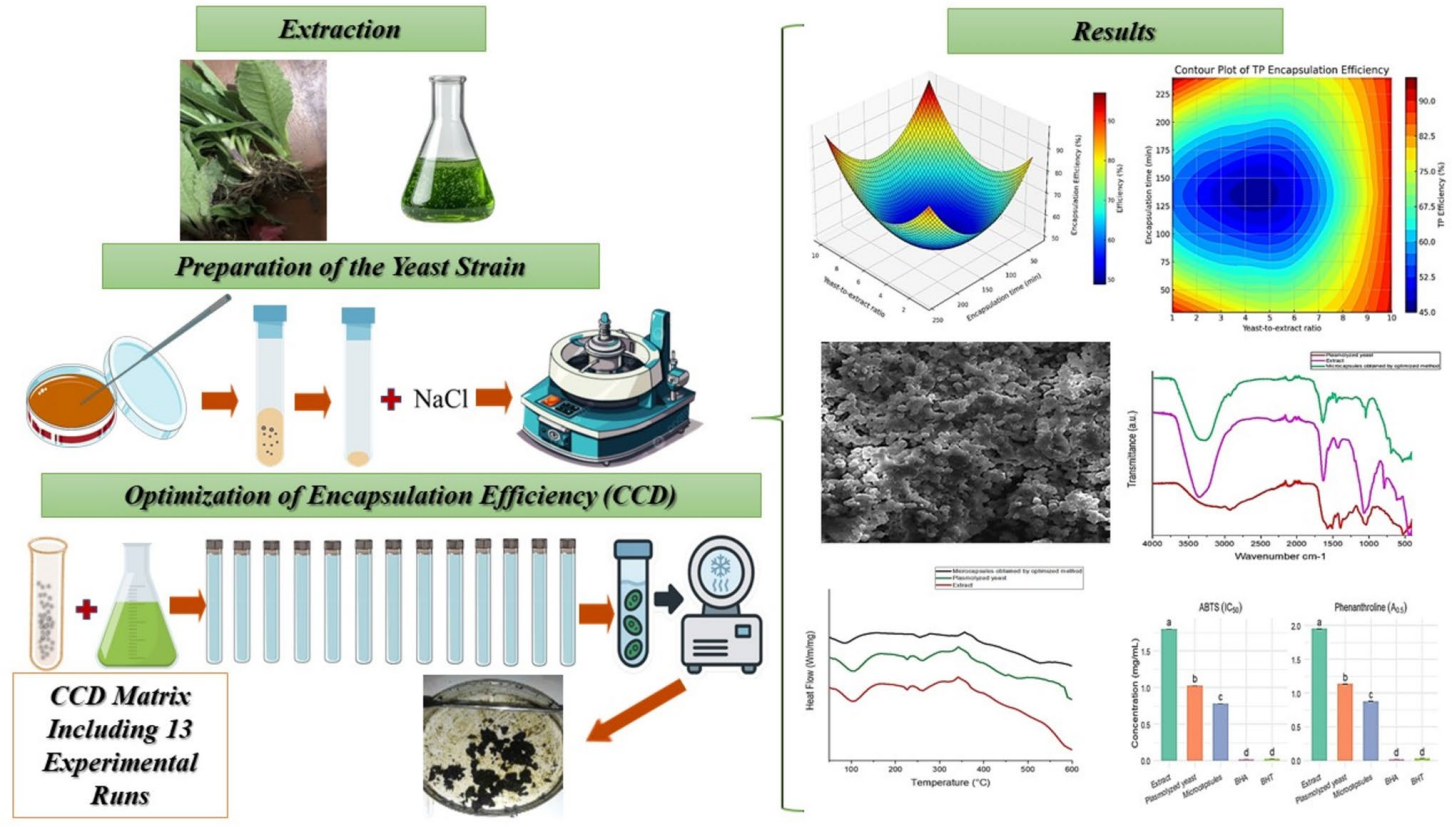

**Supplementary Information:**

The online version contains supplementary material available at 10.1007/s10068-026-02128-6.

## Introduction

For centuries, medicinal plants have played a central role in the treatment and prevention of human diseases, forming the cornerstone of many traditional healing systems. This is largely due to their richness in secondary metabolites—such as polyphenols, alkaloids, terpenes, and flavonoids—which exhibit a broad spectrum of biological activities. According to the World Health Organization (WHO), nearly 80% of the global population continues to depend on traditional medicine as a primary source of healthcare (Alanazi et al. 2023; Garg et al. 2021).

Algeria, endowed with exceptional biodiversity and a rich reservoir of medicinal and aromatic plants, represents a strategic reservoir for the development of natural bioactive compounds with applications in pharmaceuticals, cosmetics, perfumery, and food industries. These plants are valued not only for their therapeutic attributes but also for their distinctive aromatic properties and functional benefits. They yield essential oils and complex phytochemical mixtures with demonstrated biological efficacy (Belhouala and Benarba 2021).

Among the natural compounds attracting growing interest, plant-derived antioxidants have received particular attention due to their ability to neutralize oxidative stress, a key factor in the pathogenesis of chronic diseases (Lee et al. 2025; Pérez-Torres et al. 2021; Rapa et al. 2019). These compounds are typically extracted from leaves, roots, fruits, seeds, and other plant parts. Within this context, *Pulicaria odora*, a species belonging to the Asteraceae family, emerges as a valuable and underexplored medicinal resource. Despite its widespread traditional use, scientific efforts to valorize its bioactive profile—particularly through advanced encapsulation systems—remain limited.

*Pulicaria* is a diverse genus belonging to the Asteraceae family, encompassing an estimated 85 to 100 herbaceous species. These plants are mainly found in Europe, Asia, and Africa, with a strong presence in the Mediterranean basin, which represents a key center of diversity for the genus. Species within *Pulicaria* exhibit significant ecological and taxonomic variation, and several are endemic to specific regions (Mladenović et al. 2018). In Algeria, 16 species have been identified, including four that are endemic to the Saharan region (Boumaraf et al. 2016). *P. odora* is widely used in North African folk medicine for the treatment of digestive discomfort, menstrual cramps, back pain, wounds, and gastric ulcers (Saidani et al. 2023). It is also traditionally administered to women during the postpartum period. Additionally, its aromatic profile lends itself to culinary use, especially in the flavoring of bread and meat. Its roots are particularly prized for their anti-inflammatory effects (Boudebbaz et al. 2025; Hanbali et al. 2005).

The therapeutic efficacy of *P. odora* is mainly attributed to its richness in polyphenolic compounds with potent antioxidant properties. However, these compounds are chemically unstable and susceptible to degradation under environmental stressors such as light, oxygen, and elevated temperatures. To overcome these limitations, encapsulation technologies have emerged as a promising means of stabilizing and protecting sensitive bioactives. Encapsulation within micro- or nanostructures helps shield active molecules, extend shelf life, and enhance bioavailability and functional performance (Ozkan et al. 2020; Yin et al. 2022).

Among the various encapsulation matrices, the use of yeast cells—particularly *Saccharomyces cerevisiae*—as natural, biodegradable carriers has attracted increasing attention. This technique is low-cost, scalable, and fully compatible with food and nutraceutical applications (Günal-Köroğlu et al. 2024; Grambusch et al. 2024; Kasote et al. 2018; Labuschagne 2018; Round and Nelson 2006). The structural composition of the yeast cell wall, composed of mannoproteins, β-glucans, and chitin-like polysaccharides, offers a porous and biocompatible matrix that promotes entrapment and gradual release of bioactives while shielding them from oxidative degradation. Notably, yeast-based encapsulation eliminates the need for synthetic additives, aligning with the growing demand for clean-label and eco-friendly ingredients (Balakrishnan et al. 2025; Doolam et al. 2025; Joye and McClements 2014; Li et al. 2022; Oliveira et al. 2025; Utama et al. 2023).

Despite the promising advantages of this system, the encapsulation of phenolic compounds from Algerian endemic plants—particularly *Pulicaria odora*—within yeast cells has not yet been reported. This study represents a pioneering attempt to valorize this species through biocompatible encapsulation, contributing to the development of innovative and natural delivery systems.

To ensure the efficiency and reproducibility of the encapsulation process, optimization strategies are essential. In this context, Response Surface Methodology (RSM), and specifically Central Composite Design (CCD), offers a rigorous and systematic approach for evaluating and modeling the effects of process parameters. This statistical tool enables the identification of optimal conditions while minimizing experimental runs, making it highly suitable for formulation development in both academic and industrial settings (Bazaria and Kumar 2017).

The present study aims to optimize the encapsulation of *Pulicaria odora* extract using plasmolyzed yeast cells by investigating the effects of key factors such as the yeast-to-extract ratio and the encapsulation time. Additionally, a detailed characterization of the resulting microcapsules will be conducted, including morphological examination, particle size distribution, and evaluation of encapsulation efficiency. The overall objective is to establish a natural, effective, and scalable encapsulation system capable of preserving the integrity of plant-derived antioxidants, with potential applications in the food, pharmaceutical, and nutraceutical industries.

## Materials and methods

### Plant material

The plant used in this study is *Pulicaria odora*, belonging to the Asteraceae family. It is known for its medicinal properties, particularly its antioxidant and anti-inflammatory effects. This study aims to evaluate the impact of phenolic compound encapsulation within yeast cells on their content and antioxidant activity in *Pulicaria odora*. For this purpose, leaves and roots were selected as parts of interest and harvested on February 25, 2025, from Louloudj, Skikda, Algeria. After collection, the samples were carefully rinsed with tap water to remove debris, then placed in clean plastic bags and transported to the laboratory.

### Extraction

Polyphenols were extracted by maceration according to the method described by Ng and See (2019), with slight modifications. After harvesting *Pulicaria odora,* both aerial (leaves) and underground (roots) parts of the plant were thoroughly washed with tap water to remove surface impurities. The cleaned plant materials were then chopped into small pieces and ground in a mortar with 200 mL of ethanol to form a homogeneous mixture. This mixture was subsequently transferred to a beaker containing 800 mL of ethanol to ensure complete immersion of the plant material. The beaker was covered with aluminum foil to protect the contents from light and kept in a dark, clean environment for 24 h to facilitate polyphenol extraction.

Following maceration, the mixture was filtered to remove plant residues, and the ethanol was evaporated using a rotary evaporator to concentrate the extract. The concentrated extract was then lyophilized to obtain a dry polyphenol-rich powder.

### Microencapsulation of phenolic compounds

#### Preparation of the yeast strain

*Saccharomyces cerevisiae* ATCC 9763 was cultured on Petri dishes containing Sabouraud agar at 30 °C for 48 h. The resulting cells were used to inoculate a liquid nutrient broth adjusted to an optical density of 0.2 at 600 nm. Cultures were incubated at 30 °C for three days. After incubation, cells were harvested by centrifugation at 6000 rpm for 10 min (Nguyen et al. 2018).

Plasmolysis of yeast strains.

Forty grams of yeast were suspended in 500 mL of a 10% NaCl solution and subjected to plasmolysis at 55 °C for 24 h. After plasmolysis, cells were separated from the supernatant by centrifugation at 2000 g for 10 min. The pellet was washed with distilled water and agitated at room temperature for one hour to remove NaCl. This washing and centrifugation step was repeated twice. The purified cells were then lyophilized (Kurek et al. 2023).

#### Optimization of encapsulation efficiency

A Central Composite Design (CCD) was employed to optimize the encapsulation efficiency of polyphenols extracted from *P. odora.* Two independent variables were considered: the yeast-to-extract ratio and the encapsulation time. The dependent variable measured was the antioxidant activity of the encapsulated product. This experimental approach is based on Response Surface Methodology (RSM), a widely used technique for investigating and optimizing processes influenced by multiple factors. RSM enables the construction of a predictive model in the form of a second-order polynomial equation that describes the response as a function of the studied variables (Piasecka et al. 2024).

Encapsulation of phenolic compounds was performed using plasmolyzed yeast cells in an aqueous system. A specific mass ratio of yeast to extract was applied according to a CCD matrix comprising 13 experimental runs, with the hydromodule fixed at 1:100. The mixtures were incubated at 37 °C with continuous agitation at 200 rpm in a thermostatically controlled shaker, for durations defined by the CCD design. Upon completion of the incubation period, encapsulation efficiency was evaluated using the method described by Kalinina et al. (2022).

Encapsulation efficiency was assessed to determine the ability of plasmolyzed yeast cells to encapsulate phenolic compounds. To achieve this, 0.2 g of dried powder was extracted in 2 mL of a solvent mixture composed of ethanol, acetic acid, and water (50:8:42, v/v/v), with agitation for 30 min at room temperature. Phase separation was then facilitated by centrifugation at 3000 rpm for 10 min. Total polyphenol and flavonoid contents were quantified using spectrophotometry (Kalinina et al. 2022; Robert et al. 2010). Encapsulation efficiency (EE) was calculated using the following formula:

$$EE\% = \frac{TPCe}{{TPCt}} \times 100$$

where:

- TPCₑ: Total concentration of phenolic compounds (polyphenols or flavonoids) effectively encapsulated within the yeast cells.
- TPCₜ: Initial concentration of phenolic compounds (polyphenols or flavonoids) in the extract before encapsulation

### Mathematical model 

Second-order (quadratic) models are commonly used in RSM to describe relationships between independent and dependent variables when curvature exists in the response surface. The general form of the second-degree polynomial model is:

$$Y = a0 + \mathop \sum \limits_{i = 1}^{k} aiXi + \mathop \sum \limits_{i = 1}^{k} aii Xi^{2} + \mathop \sum \limits_{i < j}^{k} aijXiXj + \varepsilon$$

where:

Y: predicted response (antioxidant activity),

a0: intercept,

ai : linear coefficients,

aii: quadratic coefficients,

aij:interaction coefficients,

Xi,Xj: coded independent variables

ε is the residual error (Goupy 2006; Laib et al. 2023; Saeed et al. 2024).

### Variable levels and experimental design

Each factor was studied at three coded levels: − 1, 0, and + 1. To ensure rotatability and orthogonality of the design, the axial distance (α\alphaα) was set to 1.414. The experimental matrix consisted of:

Factorial points at levels (−1, +1),

Axial (star) points at (±α\alphaα, 0),

Central points at (0, 0), replicated to estimate experimental error.

The total number of experiments n is defined as:

$$n = nf + ne + n0$$

where:

nf: number of factorial points,

ne: number of axial points,

n0: number of central points

The relationship between the coded values (Xi) and actual values (Ai) of the independent variables is given by:

$$Xi = \frac{Ai - A0}{{\Delta A}}$$

where:

Ai: actual value of the variable,

A0: actual value at the center point,

ΔA: step size corresponding to the distance between levels ±1 (Goupy 2006).

### Design analysis

Experimental data were analyzed using Response Surface Methodology (RSM) implemented in R software (version 4.3.2; R Core Team, 2023). This approach was employed to model the response variables—namely total polyphenol and flavonoid contents—as functions of the yeast-to-extract ratio and encapsulation time, considering their linear, quadratic, and interactive effects.

The analysis generated predictive second-order polynomial equations along with graphical representations, including two-dimensional (2D) contour plots and three-dimensional (3D) response surface plots, to visualize the relationships between the variables and to determine optimal processing conditions.

The contribution of each independent factor was quantified through regression coefficients, and their statistical significance was determined using Student’s *t*-test and associated *p*-values. Model adequacy and predictive performance were assessed through analysis of variance (ANOVA), with models considered statistically significant at p ≤ 0.05. Goodness-of-fit was further evaluated using the coefficient of determination (*R*^*2*^) and the adjusted *R*^*2*^, ensuring that the models reliably captured the variability in the experimental data.

### Quantification of phenolic compounds

The quantification of polyphenols and flavonoids was carried out before and after encapsulation in order to calculate the encapsulation efficiency for each of the 13 experimental runs defined by the CCD design.

#### Total polyphenol content

Total polyphenol content was determined using the Folin–Ciocalteu method, which involves the reduction of a phosphotungstic–phosphomolybdic acid complex by phenolic compounds, forming a blue complex measurable between 725 and 750 nm. For *Pulicaria odora* extracts, 2 mL of extract was mixed with 0.2 mL of Folin–Ciocalteu reagent and 1.4 mL of 7.5% sodium carbonate (Na_2_CO_3_, w/v). The mixture was incubated for 2 h at room temperature, protected from light. Absorbance was measured at 760 nm using a UV-1800 spectrophotometer (Shimadzu). Results were expressed as milligrams of gallic acid equivalents per gram of dry extract (mg GAE/g DE), based on a calibration curve prepared with gallic acid (Waterhouse 1999).

#### Total flavonoid content

Flavonoid quantification was performed based on the method described by Chang et al. (2002), with modifications. The method relies on the formation of a stable yellow complex between aluminum ions (Al^3^⁺) and the hydroxyl groups of flavonoids, with maximal absorbance at 415 nm. For each assay, 0.2 mL of extract was mixed with 1.72 mL of 96% ethanol and 0.4 mL of 10% aluminum chloride (AlCl₃). The mixture was agitated and incubated in the dark for 30 min at room temperature. Absorbance was recorded at 415 nm using a UV-1800 spectrophotometer (Shimadzu). Results were expressed as milligrams of quercetin equivalents per gram of dry extract (mg QE/g DE), based on a quercetin standard curve.

Physicochemical characterization of capsules obtained under optimized conditions.

### Scanning electron microscopy (SEM) analysis

The surface morphology and internal microstructure of the optimized microcapsules were examined using a field emission scanning electron microscope (FE-SEM, Quanta 250 FEG, FEI, Netherlands). Samples were carefully mounted on aluminum stubs using double-sided carbon adhesive tape and coated with a thin layer of gold using a sputter coater to enhance surface conductivity. Imaging was performed under high vacuum conditions at an accelerating voltage ranging from 10 to 20 kV.

### FTIR analysis

Fourier-transform infrared spectroscopy in attenuated total reflectance mode (ATR-FTIR) was performed to analyze *Pulicaria odora* extracts before and after optimized encapsulation of polyphenols and flavonoids. Measurements were conducted using a Cary 600 FTIR spectrometer equipped with a single-reflection diamond ATR accessory (MIRacle™, PIKE Technologies). The analyzed samples included free phenolic extracts, yeast cells (intact and plasmolyzed), and encapsulated extracts within both intact and plasmolyzed yeast cells. Spectra were acquired by averaging 32 scans in the range of 4000–400 cm⁻^1^ with a spectral resolution of 4 cm⁻^1^ (Sala et al. 2020).

### Differential scanning calorimetry (DSC) analysis

Thermal properties of the optimized encapsulated and non-encapsulated samples were evaluated using Differential Scanning Calorimetry (DSC). Approximately 10 mg of each sample was accurately weighed and sealed in standard aluminum pans. The thermal analysis was performed using a DSC Q2000 (TA Instruments, New Castle, DE, USA). Samples were heated from − 80 to 300 °C at a rate of 10 °C/min under a constant nitrogen flow (20–50 mL/min) to prevent oxidative degradation. An empty sealed aluminum pan was used as a reference. Thermal transitions, including glass transition temperature (Tg), and possible decomposition events, were recorded. These measurements provided insights into the impact of optimized encapsulation on the thermal stability and structural integrity of the phenolic compounds.

### Antioxidant activity of optimized capsules

The antioxidant potential of *Pulicaria odora* extracts and their optimized encapsulated forms was assessed using two validated spectrophotometric assays: the ABTS^●^⁺ radical cation decolorization assay and the phenanthroline-based ferric reducing antioxidant power assay.

*ABTS Assay*: This method is based on the ability of antioxidants to scavenge the ABTS^●^⁺ radical cation, resulting in a decrease in the blue-green coloration measurable by spectrophotometry. ABTS^●^⁺ is generated by oxidizing ABTS with potassium persulfate (K₂S₂O₈). Upon interaction with antioxidants, ABTS^●^⁺ is reduced, and the color fades (Re et al. 1999).

To prepare the stock ABTS solution, 19.2 mg of ABTS and 3.3 mg of K₂S₂O₈ were dissolved in 5 mL of distilled water and incubated in the dark at room temperature for 16 h. The resulting solution was diluted with distilled water to obtain an absorbance of 0.70 ± 0.02 at 734 nm. For the assay, 1 mL of this ABTS solution was added to different dilutions of the extract. After 30 min of incubation in the dark, absorbance was measured at 734 nm using a UV-1800 spectrophotometer (Shimadzu).

Antioxidant activity was calculated using the following equation:

$$\% {\text{ inhibition}} = \frac{{A {\mathrm{control}} - A{\mathrm{sample}}}}{{A {\mathrm{control}}}} \times 100$$

The IC_50_ value (the concentration causing 50% inhibition) was determined to evaluate antioxidant efficacy. Results were compared with standard antioxidants BHA and BHT.

*Phenanthroline reduction method:* The reducing power was assessed using the phenanthroline method described by Szydłowska-Czerniak (2010). Briefly, 1 mL of extract (0–1 mg/mL) was mixed with 5 mL of 0.2% ferric chloride (FeCl_3_), 3 mL of 0.5% phenanthroline, and 11 mL of methanol. The mixture was incubated at room temperature in the dark for 20 min. The resulting orange-red solution was measured at 510 nm using a UV-1800 spectrophotometer (Shimadzu). Butylated hydroxyanisole (BHA) and butylated hydroxytoluene (BHT) were used as positive standards. Results were expressed as A₀.₅ (mg/mL), representing the concentration of extract required to reach an absorbance of 0.50.

### Statistical analysis

Means and standard deviations of triplicates as well as graphical representations were computed using Excel and Origin. One-way ANOVA followed by Tukey’s post hoc test was performed using RStudio (version 2023.06.1 + 524, RStudio PBC, Boston, MA, USA) with R software (version 4.3.1, R Core Team, Vienna, Austria) to compare the mean values. Superscript letters (a, b, c, d) indicate statistically significant differences at *p* < 0.05. FTIR spectra were plotted using Origin Pro 2019 SR1 (version 9.61.0000, OriginLab Corporation, Northampton, MA, USA).

## Results and discussion

Table 1 summarizes the encapsulation efficiency of total polyphenols and flavonoids obtained from the 13 experimental runs of the Central Composite Design (CCD) optimization plan.

**Table 1:** CD matrix for assessing the impact of yeast-to-extract ratio and encapsulation time on polyphenol and flavonoid content and encapsulation efficiency

Trial	Coded X1	Coded X2	TP before encapsulation (mg GAE/g)	TP after encapsulation (mg GAE/g)	EE TP (%)	TF before encapsulation (mg QE/g)	TF after encapsulation (mg QE/g)	EE TF (%)
1	− 1	− 1	18.00	16.20	90.00	17.50	14.18	81.00
2	− 1	+ 1	18.00	16.92	94.00	17.50	14.70	84.00
3	+ 1	− 1	18.00	16.38	91.00	17.50	15.05	86.00
4	+ 1	+ 1	18.00	16.03	89.03	17.50	15.23	87.00
5	− 1	0	18.00	10.26	57.00	17.50	9.45	54.00
6	+ 1	0	18.00	16.02	89.00	17.50	15.40	88.00
7	0	− 1	18.00	13.50	75.00	17.50	13.48	77.00
8	0	+ 1	18.00	13.32	74.00	17.50	13.65	78.00
9	0	0	18.00	8.63	47.92	17.50	6.13	35.00
10	0	0	18.00	8.64	48.00	17.50	6.13	35.00
11	0	0	18.00	8.64	48.00	17.50	6.13	35.00
12	0	0	18.00	8.63	47.92	17.50	6.13	35.00
13	0	0	18.00	8.64	48.00	17.50	6.13	35.00

*TP total polyphenol content*, TF* total flavonoid content *EE TP* encapsulation efficiency of total polyphenols*, EE TF* encapsulation efficiency of total flavonoids

### Central composite design for optimization of phenolic compound encapsulation

Table 1 summarizes the experimental matrix based on a Central Composite Design (CCD) used to evaluate the effects of two independent variables—yeast-to-extract ratio (X₁) and encapsulation time (X₂)—on the encapsulation efficiency of total polyphenols and flavonoids from *Pulicaria odora* extracts. Thirteen experimental runs were conducted, including factorial, axial, and central points, with coded and actual values for both factors. For each trial, total polyphenol (TP) and flavonoid (TF) contents were measured before and after encapsulation, and encapsulation efficiencies (%) were calculated accordingly.

### Model validation for the optimization of polyphenols encapsulation efficiency

A second-order polynomial model with interaction was developed to evaluate the influence of the yeast-to-extract ratio (X1) and encapsulation time (X2) on the retention of total polyphenols (TP) after encapsulation. The model exhibited a strong fit with an R^2^value of 0.9085 and an adjusted R^2^ of 0.8432, indicating that over 90% of the variation in TP content could be explained by the model.

The regression equation for TP is as follows:

$$Total polyphenols = 19.65 + 1.78X1 + 0.186X2 - 0.095 X_{1 }^{2} + 0.00037X_{2}^{2} - 0.00057 X1X2$$

The linear effects of both the ratio (p = 0.0113) and time (p = 0.0045) were statistically significant, as were the quadratic terms X1^2^ (p = 0.0031) and X2^2^ (p = 0.0022), demonstrating the existence of non-linear behavior. In contrast, the interaction term X1X2 was not significant (p = 0.7176), suggesting no meaningful combined effect between the two factors.

Similarly, the retention of total flavonoids (TF) was modeled using a second-order polynomial regression. The model displayed strong statistical performance with an R^2^of 0.8727 and an adjusted R^2^of 0.7818, confirming its reliability for describing the relationship between process parameters and flavonoid retention.

The corresponding regression equation is:

$$TOTAL FLAVONOIDS = 19.00 - 1.79X1 - 0.120 X2 + 0.190 X_{1 }^{2} + 0.00045X_{2}^{2} - 0.00018 X1X2$$

Both linear terms were statistically significant, with p = 0.0419for the ratio and p = 0.0072for time. Likewise, the quadratic terms X1^2^ (p = 0.0139) and X2^2^ (p = 0.0039) were highly significant, revealing a non-linear influence. As in the TP model, the interaction term was not significant (p = 0.9334).

The following equations represent the quadratic models obtained through a Central Composite Design (CCD) for optimizing the encapsulation efficiency of polyphenols (Eff-Poly) and flavonoids (Eff-Flav) as a function of two independent variables: the yeast-to-extract ratio (X1) and encapsulation time (X2).

For polyphenols, the equation:

$$Eff - Poly = 49.14 + 4.64X1 + 0.17X2 + 20.92 X_{1 }^{2} + 22.42X_{2}^{2} - 1.49 X1X2$$

shows that the main effect of the yeast-to-extract ratio (X1) is more significant than that of encapsulation time (X2), as reflected by the higher linear coefficient (4.64 vs. 0.17). Additionally, the positive coefficients of the quadratic terms (X₁^2^ and X₂^2^) indicate an upward curvature, suggesting that efficiency increases non-linearly with each factor and reaches a maximum at optimal levels. The interaction term (X1*X2) has a moderate and negative effect, implying that simultaneous increases in both variables may lead to a slight antagonistic effect, slightly reducing the efficiency.

For flavonoids, the equation:

$$Eff - Flav = 39 + 7X1 + 0.83X2 + 22 X_{1 }^{2} + 28.50X_{2}^{2} - 0.50 X1X2$$

exhibits a similar behavior, with a strong influence of the yeast-to-extract ratio (X1), as indicated by the linear coefficient (7), and a positive effect of encapsulation time (X2). The quadratic terms are clearly positive, confirming a convex surface response, which is characteristic of an optimal condition. The interaction effect is very weak (–0.50), suggesting minimal cross-influence between the two variables.

Overall, these models indicate that increasing both the yeast-to-extract ratio and encapsulation time—up to a certain threshold—significantly enhances encapsulation efficiency. The strong contribution of the quadratic terms highlights the importance of precisely adjusting both factors to achieve the optimal encapsulation performance predicted by the models.

Analysis of variance (ANOVA) was performed to assess the validity of the response surface models fitted for the encapsulation efficiency of total polyphenols and flavonoids.

For total polyphenols, the overall model was significant, with the quadratic term showing a highly significant effect (p = 0.0003), indicating a pronounced curvature in the response surface. In contrast, the linear effects and interaction term were not statistically significant (p > 0.05), suggesting that the response did not vary linearly with the studied factors nor through their simple interaction.

The coefficient of determination (R^2^) for this model was 0.91, indicating that 90.8% of the variability in the response is explained by the model. The adjusted R^2^ was 0.84, confirming the model’s robustness despite a relatively limited number of experimental runs.

Similar trends were observed for flavonoids, where only the quadratic term was significant (p = 0.0009), while the linear and interaction effects were not significant. The model exhibited an R^2^ of 0.87 and an adjusted R^2^of 0.86, reflecting an excellent predictive capability.

These results confirm that variations in encapsulation efficiency are primarily influenced by quadratic effects, justifying the use of a second-order polynomial model. The obtained response surfaces highlight a well-defined optimal region, characterized by significant curvature and the absence of notable interaction between the two studied factors.

### Response surface analysis of encapsulation efficiency

**Fig. 1:**
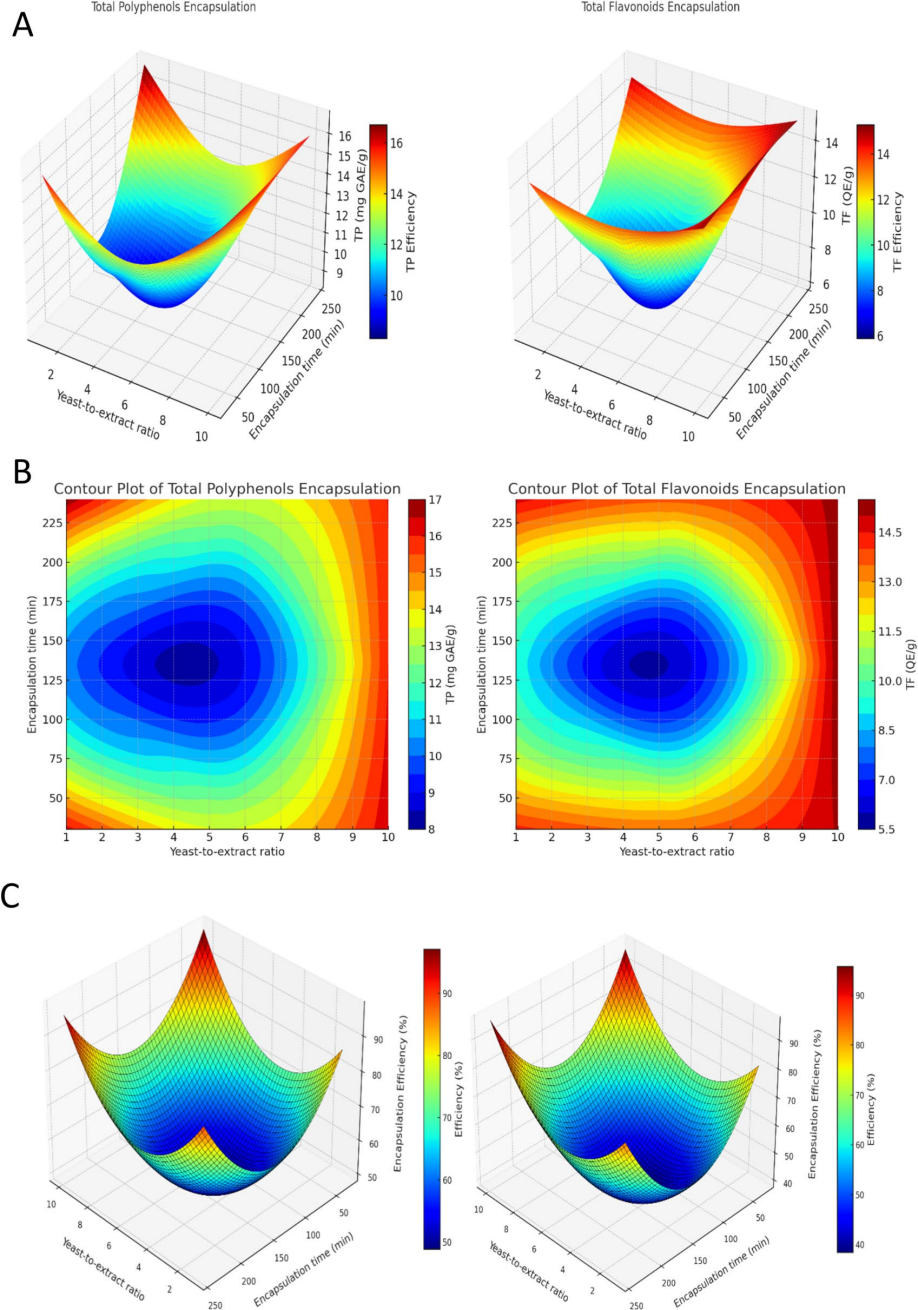

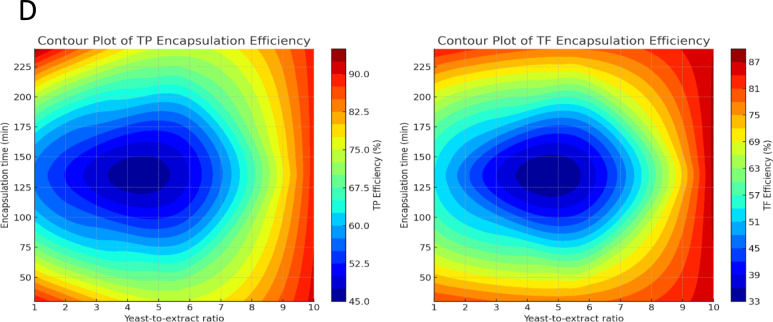
Response surface and contour plots for total polyphenol and flavonoid content and encapsulation efficiency (**A**) 3D response surface for total polyphenol (TP) and total flavonoid (TF) content after encapsulation, (**B**) 2D contour plots for TP and TF content after encapsulation, (**C**) 3D response surface for TP and TF encapsulation efficiency, (**D**) 2D contour plots for TP and TF encapsulation efficiency

Figure 1 presents the response surface and contour plots derived from the central composite design (CCD), highlighting the interaction effects between encapsulation time and yeast-to-extract ratio on both the content and encapsulation efficiency of total polyphenols and flavonoids. The surfaces illustrate how each variable, individually and in combination, influences the responses. The inclusion of polyphenol and flavonoid content as response variables provides further insight into the extract retention capacity of the system. The 2D contour projections facilitate the identification of optimal regions for maximizing both bioactive compound content and encapsulation performance.

The post-encapsulation content of total polyphenols (TP) and total flavonoids (TF) varied significantly according to both the yeast-to-extract ratio and the incubation time, displaying characteristic bell-shaped trends at lower and intermediate ratios.

For polyphenols, at low (1) and intermediate (5.5) yeast-to-extract ratios, TP levels were initially high (e.g., ~ 16.2 mg GAE/g at 30 min for ratio 1), dropped markedly at intermediate times (~ 10.26 mg GAE/g at 135 min), and then rose again at prolonged durations (up to ~ 16.92 mg GAE/g at 240 min for ratio 1). This transient decrease may reflect a temporary loss of loosely bound polyphenols or a redistribution process within the yeast matrix. In contrast, at the highest ratio (10), TP content remained both high and stable across all incubation times, with values ranging between 16.02 and 16.38 mg GAE/g. This indicates that a higher number of available binding sites prevents desorption and supports the sustained retention of polyphenols throughout the encapsulation process.

Flavonoid content after encapsulation followed a similar time- and ratio-dependent profile. At ratios of 1 and 5.5, flavonoid levels peaked early (e.g., ~ 14.18 mg QE/g at 30 min for ratio 1), declined substantially at intermediate time points (~ 9.45 mg QE/g at 135 min), and increased again toward the end of incubation (~ 14.70 mg QE/g at 240 min). This dynamic suggests a desorption–reassociation mechanism, where flavonoids initially bind rapidly, then partially detach, before reintegrating into the yeast matrix as equilibrium conditions stabilize. At a ratio of 10, TF values remained consistently high and unaffected by incubation time, ranging from 15.05 to 15.40 mg QE/g. These results confirm the influence of yeast concentration on improving the retention capacity and point to its buffering role against temporal fluctuations in flavonoid content.

When encapsulation efficiency is considered, a complementary pattern emerges. For polyphenols, efficiency was strongly dependent on time and yeast concentration. At ratios of 1 and 5.5, an inverted bell-shaped (U-shaped) response was observed: initially high efficiencies dropped significantly at intermediate times (reaching ~ 58% and ~ 50%, respectively), then increased slightly with prolonged incubation. This pattern may result from a temporary imbalance between rapid initial adsorption and subsequent desorption of weakly bound compounds. With time, molecular rearrangements likely facilitate the reattachment of polyphenols to deeper or more stable sites within the yeast structure. Conversely, at the highest ratio (10), encapsulation efficiency remained stable and high (> 90%) throughout, indicating rapid and effective site saturation that minimizes compound loss.

A parallel behavior was observed for flavonoid encapsulation. Again, at ratios of 1 and 5.5, the efficiency curve displayed a U-shape, with a marked decline at intermediate times (dropping below 40% for ratio 5.5 at 135 min). This greater sensitivity may be attributed to slower diffusion kinetics or weaker binding affinities of flavonoids—often due to their larger size, glycosylation, or lipophilic nature. Over extended incubation, these molecules may progressively migrate and integrate into deeper layers of the yeast cell wall or membrane, resulting in improved retention. At a ratio of 10, TF encapsulation efficiency remained high (> 88%) and independent of time.These observations demonstrate that for both compound classes, a high yeast concentration enhances encapsulation efficiency, but a sufficiently long contact time is required to overcome transient desorption phenomena and promote stabilization of molecular interactions.

The inverted bell-shaped profile may also be interpreted in light of the yeast-cell encapsulation mechanism, which occurs in several successive steps: adsorption of bioactive molecules onto the cell surface, permeation through the wall, membrane penetration, and finally intracellular distribution following membrane disorganization (Dadkhodazade et al. 2021). Initially, adsorption is driven by the natural affinity of biomolecules for the cell surface (Lefebvre et al. 2021). Moreover, increasing the yeast-to-extract ratio significantly enhances encapsulation due to the greater number of binding sites present in the cell wall, which is rich in β-glucans, mannoproteins, and chitosan-like polysaccharides that interact through hydrogen bonding, π–π stacking, and ionic interactions (Fu et al. 2022; Karaman 2021; Semouma et al. 2024). Once these adsorption sites are occupied, efficiency tends to decrease. As the second step—permeation through the cell wall—progresses, sites are released, and efficiency increases. This step is considered rate-limiting due to the absence of active transporters (De Nobel and Barnett 1991) and thus occurs via passive diffusion, primarily from adsorbed molecules, driven by a steep concentration gradient between the adsorbed droplets and the periplasmic space, eventually leading to intracellular distribution.

The encapsulation kinetics are often biphasic: a rapid initial phase (< 10 min) corresponding to adsorption and membrane swelling, followed by a slower phase (several hours) associated with intracellular redistribution of the compounds (Dadkhodazade et al. 2021). Finally, the use of plasmolyzed yeast cells, obtained through osmotic stress, can increase the available intracellular space without compromising the integrity of the wall, thereby facilitating compound entrapment (Dong et al. 2024; Shi et al. 2007). However, this strategy does not eliminate the need for prolonged contact time, which is especially critical for flavonoids.

### Prediction of optimal conditions using the response surface model

The response surface model was used to determine the theoretical optimal conditions for maximizing both the encapsulation efficiency and retention of polyphenols and flavonoids.

For total polyphenols (TP), the model predicts a maximum encapsulation efficiency of 95.83%, with a corresponding TP content of 17.72 mg GAE/g of dry extract, achieved at a yeast-to-extract ratio of 10 and an incubation time of 240 min. Similarly, for total flavonoids (TF), the optimal conditions—also at a 10:1 ratio and 240 min—yield an encapsulation efficiency of 96.83% and a TF content of 16.95 mg QE/g of dry extract (Figure S1).

These results confirm that although initial adsorption occurs rapidly, optimal encapsulation and compound retention require a combination of high yeast concentration and prolonged incubation time. Such conditions facilitate not only surface binding but also deeper diffusion, access to internal binding domains, and the stabilization of molecular interactions within the yeast matrix. The strong agreement between predicted and experimental values reinforces the validity of the model and its relevance for guiding process optimization in yeast.

based encapsulation systems.

### Microscopic evidence of yeast cell structural modifications during the encapsulation of *Pulicaria odora* extracts

**Fig. 2:**
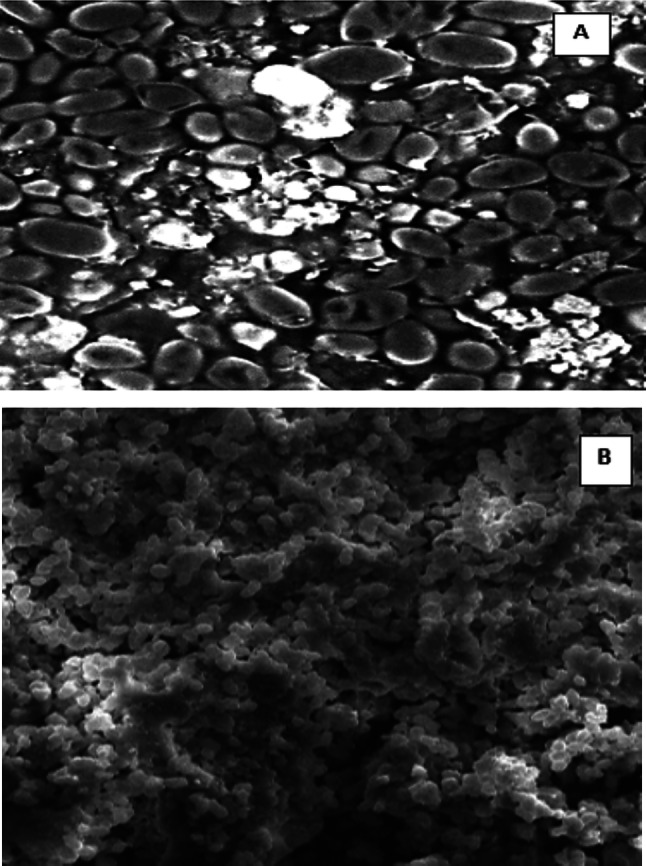
Microscopic images illustrating the encapsulation of *Pulicaria odora* extracts by yeast cells: **A**) Yeast cells prior to encapsulation; **B**) Microcapsules obtained after optimization of the encapsulation process

Figure 2 presents scanning electron microscopy (SEM) images illustrating the encapsulation process of *Pulicaria odora* extracts by yeast cells. Image A shows yeast cells prior to encapsulation, displaying a typical oval morphology with smooth, well-defined surfaces and clearly individualized cells. In contrast, Image B reveals the formation of microcapsules following process optimization. The microcapsules exhibit a dense, compact, and granular structure, with cell agglomeration and the loss of visible individual contours, suggesting successful adsorption and integration of the plant extract.

This morphological transformation is likely attributable to the adsorption step of bioactive compounds onto the yeast cell surface. Similar cellular agglomerates have been reported by Paramera et al. (2011), who associated them with the formation of interfacial hydrophobic layers. Adsorption is known to be an exothermic process, with maximum efficiency typically occurring below 35 °C (Aksu and Tezer 2000). While extensively studied in the context of soluble pollutants such as heavy metals and dyes (Aksu and Dönmez 2003; Aksu 2005; Göksungur et al. 2005; Guler and Sarioglu 2014), the same principles apply to hydrophobic bioactive compounds.

These molecules may diffuse through the cell wall and accumulate in the lipid bilayer of the plasma membrane, particularly when their logP ranges between 2 and 3. This leads to the formation of a thermodynamic “sink.” However, excessive accumulation can disrupt the membrane’s integrity, a phenomenon previously observed in encapsulated yeasts (Dadkhodazade et al. 2021). The resulting membrane rupture facilitates the redistribution of hydrophobic compounds into intracellular lipid structures, while also hindering diffusion due to the expansion of the periplasmic space. This sequence of events likely contributes to the clustered and disorganized appearance of yeast cells observed post-encapsulation.

### FTIR analysis

**Fig. 3:**
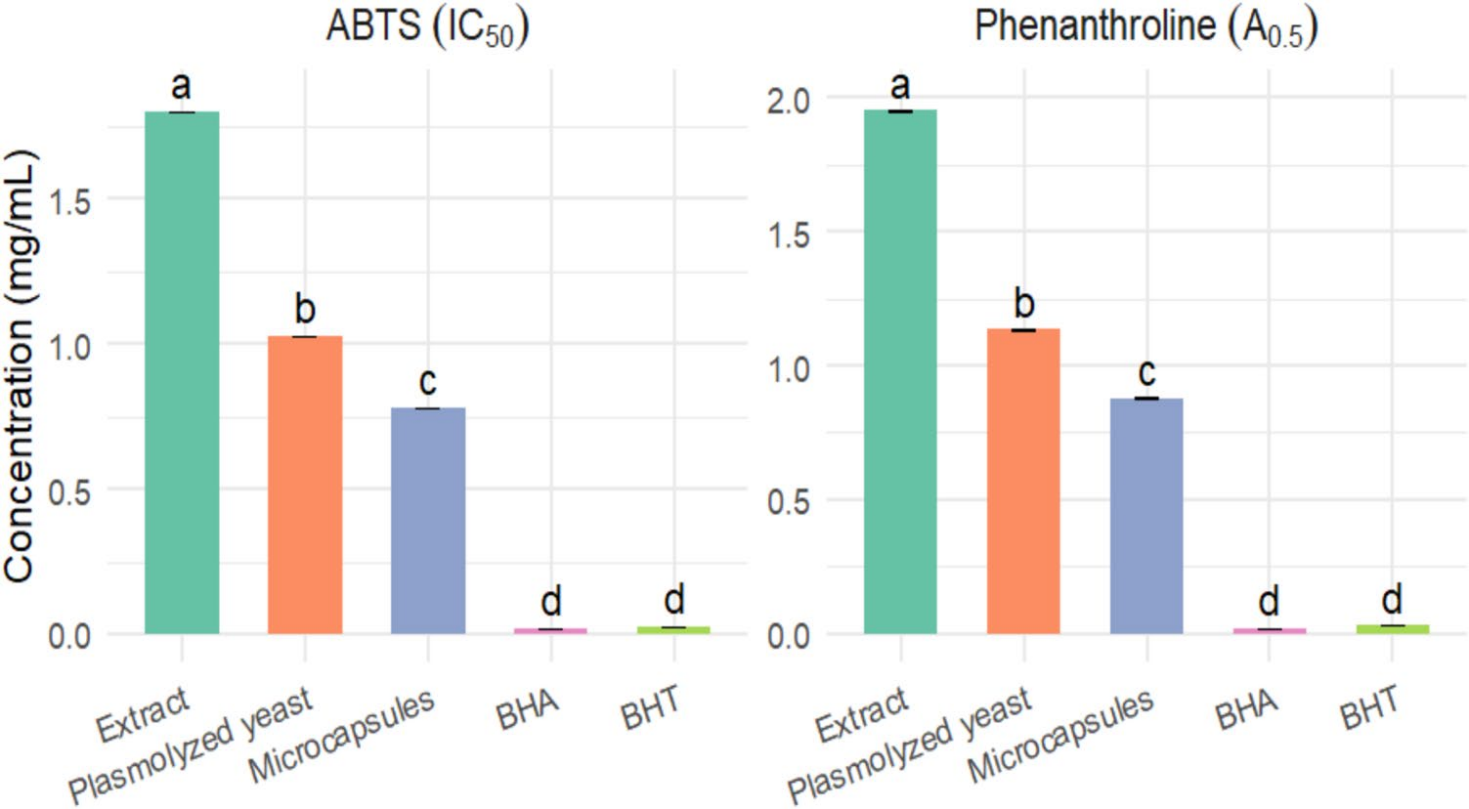
Antioxidant activity measured by ABTS and Phenanthroline assay

Figure 3 presents the FTIR spectral profiles of the key constituents involved in the encapsulation system, highlighting their distinct chemical fingerprints and confirming the successful integration of *Pulicaria odora* extract into the yeast matrix. The FTIR spectrum of plasmolyzed yeast exhibits characteristic absorption bands around 3300 cm⁻^1^, attributed to O–H and N–H stretching vibrations, as well as peaks at 2920–2850 cm⁻^1^, corresponding to aliphatic C–H stretching. A pronounced amide I band near 1650 cm⁻^1^ indicates the presence of proteins, while other peaks associated with polysaccharides reflect the biochemical complexity of the yeast cell wall (Saroglu et al. 2023).

The spectrum of the *P. odora* extract reveals strong absorption bands around 3300 cm⁻^1^ (O–H stretching), 1600 and 1500 cm⁻^1^ (C = C aromatic stretching), and approximately 1100 cm⁻^1^ (C–O stretching), which are characteristic of phenolic compounds and flavonoids (Chacón-Figueroa et al. 2025). These bands confirm the polyphenolic richness and functional group diversity of the plant extract.

In the spectrum of the encapsulated system, absorption bands originating from both the yeast and the extract are clearly observed. Notably, shifts in peak positions and changes in intensity, particularly in the 1600–1000 cm⁻^1^ region, suggest molecular interactions and the formation of hydrogen bonds between bioactive compounds and functional groups within the yeast cell wall (Kalinina et al. 2024a, b; Semouma et al. 2024).

Furthermore, the emergence of new absorption patterns and band merging within this region implies the establishment of non-covalent interactions, likely driven by electrostatic forces and hydrogen bonding. These interactions contribute to the stabilization of the bioactive compounds, while also suggesting entrapment within the porous structure of the plasmolyzed yeast, rather than mere surface adsorption. Overall, FTIR analysis supports the hypothesis that encapsulation occurred through both physical entrapment and molecular affinity between the plant extract and yeast constituents. This structural compatibility is a critical factor for the stability, retention, and controlled release of bioactives in food or pharmaceutical applications.

### Differential scanning calorimetry (DSC) analysis

The thermal behavior of the samples was investigated using Differential Scanning Calorimetry (DSC), and the resulting thermograms are presented in Figure S2. An initial endothermic event, observed between 50 and 150 °C in all samples, is attributed to the evaporation of residual moisture (Paramera et al. 2011). This phenomenon is more pronounced in the free extract, suggesting weak interactions between water molecules and the surrounding matrix. In contrast, the presence of the cellular matrix in the encapsulated samples appears to improve moisture retention.

Between 200 and 400 °C, the thermograms reveal major thermal transitions (Figure S3). The free extract displays a sharp endothermic peak around 312 °C, likely corresponding to a glass transition (Tg), followed by melting or structural disorganization of bioactive components, particularly phenolic compounds and fatty acids. This transition is shifted to higher temperatures in the plasmolyzed yeast sample (≈ 341.67 °C), indicating a certain degree of thermal stabilization provided by the cellular structure. More notably, the microcapsules obtained via the optimized method exhibit a significant shift in the main thermal peak beyond 357 °C, accompanied by a smoother and less abrupt profile. This reflects effective thermal protection conferred by the encapsulating matrix, which limits the molecular mobility of the encapsulated compounds and delays their degradation.

Beyond 400 °C, a marked decrease in heat flow is observed, corresponding to the thermal decomposition or pyrolysis of organic constituents. This degradation occurs more rapidly and intensely in the free extract, while it is significantly reduced in the encapsulated forms. The thermogram of the optimized microcapsules shows enhanced thermal stability, maintaining integrity up to nearly 550 °C, thus confirming that the encapsulation process provides an effective barrier against thermal degradation (Karaman 2021).

Overall, these results demonstrate that the optimized encapsulation technique significantly improves the thermal stability of bioactive extracts. Similar findings were reported by Paramera et al. (2011), who showed that yeast cells provided better thermal protection for curcumin between 100 and 200 °C compared to β-cyclodextrin and modified starch. Likewise, Normand et al. (2005) reported that yeast cell walls remain stable up to 263 °C and effectively protect encapsulated limonene. Semouma et al. (2024) also observed enhanced thermal stability of myrtle extracts following encapsulation in both intact and plasmolyzed yeast cells. Furthermore, Young and Nitin (2019) demonstrated that plasmolyzed yeast cells protected curcumin from degradation at 70 °C for 30 min and at 90 °C for 1 min, due to structural modifications that enhance binding to curcumin. In addition, Lieu et al. (2020) found that combining ethanol and ultrasound treatments reduced thermal losses of anthocyanins during encapsulation. Collectively, these studies support the conclusion that yeast cells significantly enhance the thermal resistance of microencapsulated bioactive compounds.

### Effect of encapsulation on the antioxidant activity of *Pulicaria odora* extracts

Figure 3 presents the antioxidant activity of the various samples, evaluated using the ABTS and phenanthroline assays and expressed as IC_50_ and A_0.5_ values, respectively. The free extract exhibited the lowest antioxidant capacity, with an IC_50_ of 1.800 ± 0.002 mg/mL and an A_0.5_ of 1.950 ± 0.001 mg/mL, indicating limited effectiveness in radical scavenging. Interestingly, the empty plasmolyzed yeast cells also demonstrated measurable activity (IC_50_ = 1.028 ± 0.001 mg/mL; A_0.5_ = 1.134 ± 0.001 mg/mL), likely due to intrinsic components of the yeast cell wall with reductive properties. In contrast, the microcapsules obtained using the optimized method, which encapsulate the extract, exhibited substantially enhanced antioxidant activity (IC_50_ = 0.780 ± 0.001 mg/mL; A_0.5_ = 0.880 ± 0.001 mg/mL), highlighting the efficiency of encapsulation in preserving and enhancing the bioactivity of the extract. However, this activity remains lower than that of the reference antioxidants BHA and BHT, which showed the lowest IC_50_ and A_0.5_ values in both assays, confirming their strong radical-scavenging potential.

These findings are in line with several previous studies on the encapsulation of phenolic compounds using yeast cells, which have reported a marked improvement in antioxidant activity following microencapsulation. In particular, the ABTS results agree with those of Hatip and Karaman (2025), who demonstrated that encapsulation—whether using intact or plasmolyzed yeast cells—significantly enhances the antioxidant capacity of plant extracts. These results reinforce the potential of *Saccharomyces cerevisiae* as an effective biological carrier for the delivery of plant-derived bioactive compounds.

Among the various encapsulation strategies, the use of plasmolyzed yeast cells appears particularly advantageous. Several studies have reported that this form improves both the stability and bioavailability of antioxidants during digestion, with preservation rates reaching up to 80% compared to non-encapsulated forms (Gümüşay et al. 2025). Additionally, using plasmolyzed yeast as a delivery system has been shown to increase the bioavailability of plant polyphenols by 30–40%, further enhancing their biological activity.

Therapeutic applications of yeast-based encapsulation systems have also begun to emerge. For instance, nobiletin-loaded yeast microcapsules were found to effectively reduce oxidative stress, inhibit NLRP3 inflammasome activation, and modulate immune responses in patients with ulcerative colitis, offering a novel strategy for treating inflammation-related intestinal disorders (Kalinina et al. 2024a, b; Yang et al. 2024).

Similarly, Semouma et al. (2024) reported that encapsulation of plant extracts in plasmolyzed yeast cells resulted in significantly higher antioxidant activity compared to intact yeast, as evaluated by five different assays (DPPH, ABTS, ferric reducing power, o-phenanthroline, and silver nanoparticle reduction). Chacón-Figueroa et al. (2025) also observed strong DPPH radical scavenging activity in yeast-based microcapsules. Furthermore, plasmolyzed yeast cells have demonstrated the capacity to encapsulate a wide range of plant antioxidants—such as dihydroquercetin, rutin, curcumin, and myricetin—achieving encapsulation efficiencies ranging from 57 to 64%, particularly when polyphenols are nanostructured (Kalinina et al. 2024a, b).

The enhanced antioxidant activity observed in our study may therefore result from the intrinsic activity of the plant extract, the yeast matrix, or more likely, from a synergistic interaction between bioactive compounds and the yeast cell structure. Polyphenols and flavonoids are well-known for their potent antioxidant properties, and encapsulating them can not only stabilize these compounds but also enhance their efficacy by protecting them from degradation.

Moreover, yeast cells themselves possess intrinsic antioxidant activity. In particular, β-glucans and mannans—key components of the yeast cell wall—exhibit moderate hydroxyl radical scavenging capacity, although their activity against DPPH radicals is generally lower (typically below 15%). However, chemically modified derivatives such as carboxymethylated β-glucan (CM-glucan) have been shown to display significantly enhanced antioxidant activity, reaching up to 65% against hydroxyl radicals (Machová and Bystrický 2013; Machová et al. 2014). This effectiveness is closely linked to the susceptibility of these polysaccharides to oxidative degradation by free radicals (Cerit 2025; Laib et al. 2021).

In addition to polysaccharides, yeast-derived protein fractions also contribute to antioxidant activity. Innovative approaches such as ultrasound-assisted Maillard conjugation between spent yeast protein hydrolysates and polysaccharides from agro-industrial by-products (e.g., aronia pomace) have proven capable of enhancing antioxidant activity and effectively encapsulating bioactives, thereby contributing to sustainable food production systems (Kalinina et al. 2022).

Finally, the overall antioxidant effect of yeast-based systems is not limited to their encapsulation capabilities. It also stems from the intrinsic presence of bioactive compounds within yeast cells—such as glutathione—which actively participate in radical scavenging and contribute to the stabilization and enhanced performance of encapsulated polyphenols (Vargas‐Ochoa et al. 2016).

## Conclusion

This study demonstrated the successful optimization of polyphenol encapsulation from *Pulicaria odora* using plasmolyzed *Saccharomyces cerevisiae* cells, applying a statistically guided methodology. The resulting microcapsules not only showed significantly improved antioxidant activity but also exhibited enhanced thermal stability, confirming the efficiency of this natural, sustainable, and cost-effective encapsulation strategy. Structural and spectroscopic characterizations (FTIR, DSC) further supported the effective entrapment and potential interactions between phenolic compounds and the yeast matrix.

These findings highlight the relevance of yeast-derived microencapsulation systems as promising carriers for bioactive compounds, offering dual benefits of protection and targeted delivery. The biological affinity and structural compatibility between plant polyphenols and yeast cell walls open new avenues for functional food design, nutraceutical development, and pharmaceutical formulations.

More broadly, this approach contributes to the circular bioeconomy by valorizing both plant resources and microbial biomass in a green encapsulation process. Future investigations should focus on evaluating the long-term stability of the encapsulated systems, their release behavior under simulated gastrointestinal conditions, and the possible synergistic effects when combined with other natural antioxidants or essential oils. Given its scalability and versatility, this yeast-based technology holds strong potential for industrial implementation in clean-label and health-oriented products.

## Supplementary Information

Below is the link to the electronic supplementary material.

Supplementary file1 (DOCX 152 KB)

## Data Availability

All the data in the article are available from the corresponding author upon reasonable request.
