# Supplementary material for: Plasmolyzed yeast cell system for the encapsulation of Pulicaria odora polyphenols: RSM-based process optimization and functional characterization
Source: Food Sci Biotechnol. 2026 Mar 25;35(6):1467–82. doi: 10.1007/s10068-026-02128-6 (PMC13129016; doi:10.1007/s10068-026-02128-6)
Supplement: Supplementary file 1 — Supplementary file1 (DOCX 152 KB) [file 10068_2026_2128_MOESM1_ESM.docx]

**Supplementary Material**

**Plasmolyzed Yeast Cell System for the Encapsulation of *Pulicaria odora* Polyphenols: RSM-Based Process Optimization and Functional Characterization**

**Djamel Eddine Laib^1^, Imen Laib^1,2,^ Hamdi Bendif^3^*, Sulaiman A. Alsalamah^3^, Tarek H. Taha^3^, Fehmi Boufahja^3^, Walid Elfalleh^3^, Stefania Garzoli^4^***

^1^ Department of Agronomic Sciences, Faculty of Sciences, August 20, 1955 University, Skikda, Algeria. [d.laib@univ-skikda.dz](mailto:d.laib@univ-skikda.dz); [mina.laib@gmail.com](mailto:mina.laib@gmail.com);

^2^Laboratory of the Optimization of Agricultural Production in Subhumid Areas, Faculty of Sciences, University 20 August 1955, Skikda-21000, Algeria

^3^Department of Biology, College of Science, Imam Mohammad Ibn Saud Islamic University (IMSIU), Riyadh 11623, Saudi Arabia; faboufahja@imamu.edu.sa; saalsalamah@imamu.edu.sa; wbelfallah@imamu.edu.sa; hlbendif@imamu.edu.sa; [thali@imamu.edu.sa](mailto:thali@imamu.edu.sa)

^4^Department of Chemistry and Technologies of Drug, Sapienza University, P. le Aldo Moro, 5,00185 Rome, Italy. [stefania.garzoli@uniroma1.it](mailto:stefania.garzoli@uniroma1.it);

***Correspondence:** [stefania.garzoli@uniroma1.it](mailto:stefania.garzoli@uniroma1.it); [hlbendif@imamu.edu.sa](mailto:hlbendif@imamu.edu.sa)


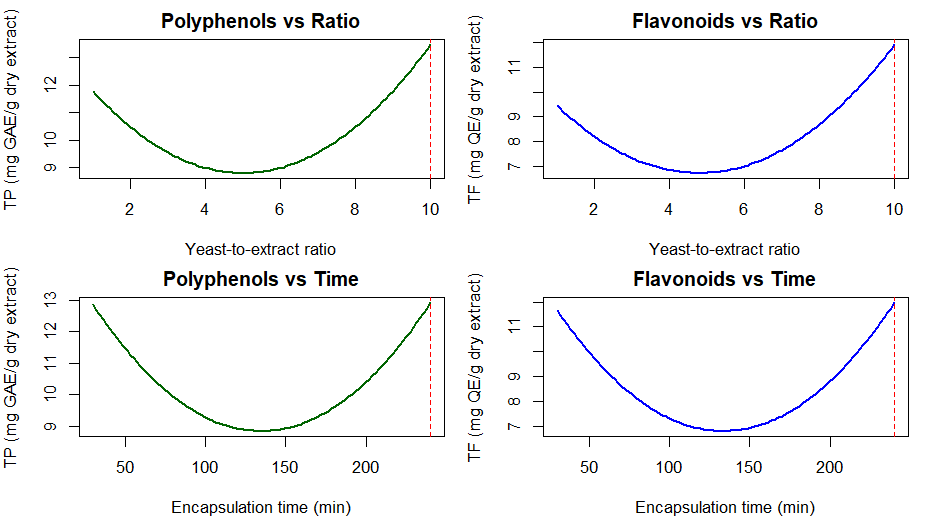


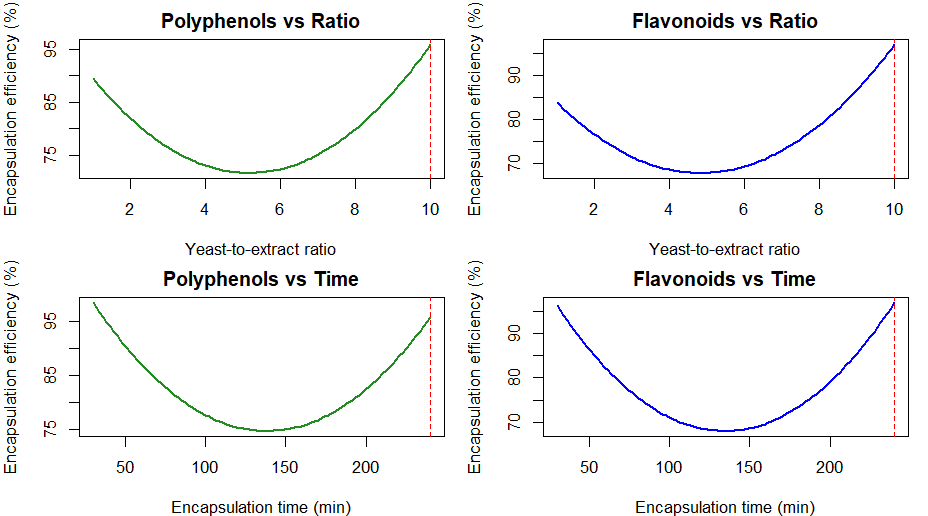
*

**Figure S1.** Model-Based Prediction of Optimal Parameters for Encapsulation Efficiency


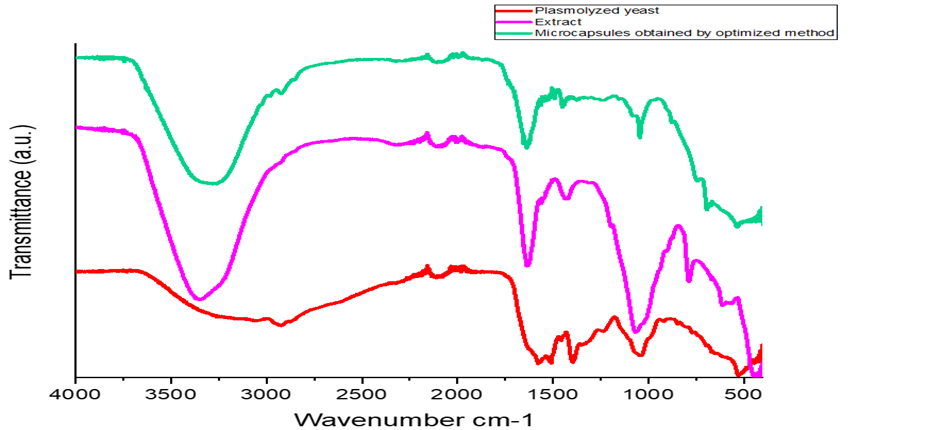


**Figure S2.** FTIR spectra of plasmolyzed yeast cells, *Pulicaria odora* extract, and the resulting microcapsules obtained through the optimized encapsulation method.

**
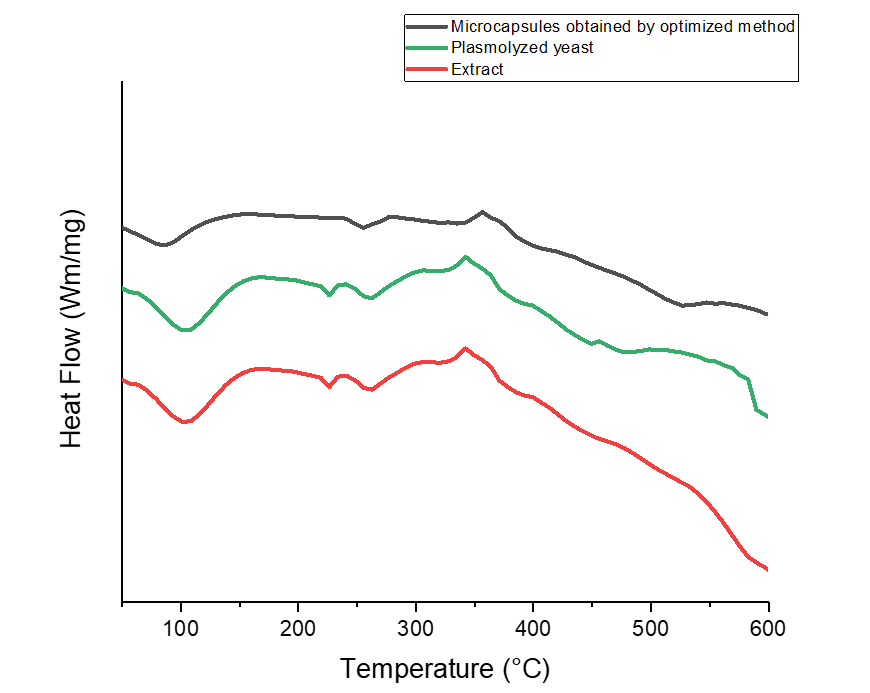
**

**Figure S3.** DSC Thermograms of Plasmolyzed Yeast, *Pulicaria odora* Extract, and Microcapsules Obtained by the Optimized Method
